# GPs' decision-making when prescribing medicines for breastfeeding women: Content analysis of a survey

**DOI:** 10.1186/1756-0500-3-82

**Published:** 2010-03-23

**Authors:** Hiranya S Jayawickrama, Lisa H Amir, Marie V Pirotta

**Affiliations:** 1 Mother & Child Health Research, La Trobe University, Melbourne, Australia; 2 Centre for Women's Health, Gender and Society, University of Melbourne, Carlton, Australia; 3 Primary Care Research Unit, Department of General Practice, University of Melbourne, Carlton, Australia

## Abstract

**Background:**

Many breastfeeding women seek medical care from general practitioners (GPs) for various health problems and GPs may consider prescribing medicines in these consultations. Prescribing medicines to a breastfeeding mother may lead to untimely cessation of breastfeeding or a breastfeeding mother may be denied medicines due to the possible risk to her infant, both of which may lead to unwanted consequences. Information on factors governing GPs' decision-making and their views in such situations is limited.

**Methods:**

GPs providing shared maternity care at the Royal Women's Hospital, Melbourne were surveyed using an anonymous postal survey to determine their knowledge, attitudes and practices on medicines and breastfeeding, in 2007/2008 (n = 640). Content analysis of their response to a question concerning decision-making about the use of medicine for a breastfeeding woman was conducted. A thematic network was constructed with basic, organising and global themes.

**Results:**

335 (52%) GPs responded to the survey, and 253 (76%) provided information on the last time they had to decide about the use of medicine for a breastfeeding woman. Conditions reported were mastitis (24%), other infections (24%) and depressive disorders (21%). The global theme that emerged was "*complexity of managing risk in prescribing for breastfeeding women"*. The organising themes were: *certainty around decision-making; uncertainty around decision-making; need for drug information to be available, consistent and reliable; joint decision-making; the vulnerable "third party" *and *infant feeding decision*. Decision-making is a spectrum from a straight forward decision, such as treatment of mastitis, to a complicated one requiring multiple inputs and consideration. GPs use more information seeking and collaboration in decision-making when they perceive the problem to be more complex, for example, in postnatal depression.

**Conclusion:**

GPs feel that prescribing medicines for breastfeeding women is a contentious issue. They manage the risk of prescribing by gathering information and assessing the possible effects on the breastfed infant. Without evidence-based information, they sometimes recommend cessation of breastfeeding unnecessarily.

## Background

Both WHO and UNICEF recommend exclusive breastfeeding for six months and continuing breastfeeding together with appropriate complementary feeding for two years or beyond [1]. During the postpartum period and thereafter, lactating women may face numerous health issues needing medicines [2,3]. In a study conducted in Brazil 96% of women received medicines in the immediate postpartum period [4]. A study among postpartum mothers in Victoria, Australia, found that 17% of women reported feeling depressed or very unhappy for more than few weeks, 42% of having backache and 14% mastitis [5]. Lactating women may also experience incidental problems like headache and musculoskeletal pain, upper respiratory tract infections (URTI), urinary tract infections (UTI) and dental problems. Proper management of such conditions is crucial for successful breastfeeding and well-being of the mother.

However, whether GPs are equipped with the proper knowledge and skills in managing such situations is a poorly researched area. In a study among GPs in Victoria, approximately 75% claimed they were confident in dealing with mastitis in the postnatal period as opposed to 39% with neck pain and 26% with postnatal depression [6]. It has been found that physicians advise against breastfeeding when prescribing certain drugs, despite established safety during breastfeeding [7].

Prescribing medicines to a breastfeeding mother may lead to untimely cessation of breastfeeding or a breastfeeding mother may be denied medicines due to the possible risk to her infant [2,8]. Both of these situations may lead to poorer outcomes for mother and/or child.

In this paper, we explore GPs' decision-making in situations where they are considering recommending or prescribing a medicine for a breastfeeding woman.

## Methods

An anonymous postal survey was conducted with general practitioners (GPs) providing shared maternity care at the Royal Women's Hospital (RWH), Melbourne, Australia, to assess their knowledge, attitudes and practices on medicines and breastfeeding (see Amir & Pirotta for more information [9]). The survey was based on items from Brodribb's questionnaire [10], the current literature and four in-depth interviews with GPs conducted by LA. A current list of GPs was obtained from the RWH (n = 666). The questionnaires consisting of closed and open ended questions and were mailed out in November 2007 and February 2008 with a cover letter and reply-paid envelope. A reminder post card was sent two weeks after the November mail out.

Relevant to this article are the last two items in the questionnaire which were open ended questions. We designed a structured question asking the participant about their last experience of using a medicine for a breastfeeding woman to elicit a free-text response with depth and robustness [11]: "Thinking about the last time you had to make a decision about use of a medicine (prescription, over-the-counter or complementary) for a breastfeeding woman, please describe - what was the situation? what did you decide? your reasons for the decision, and how did you feel about the decision-making process?" We asked GPs to report "the last time" they had to make a decision, so we could avoid GPs reporting their most difficult prescribing scenarios, reduce recall bias and also gather some information on the relative frequency of conditions requiring medicines in the postpartum. The final item was a request for further comments (identified as "Comment" in Results).

We undertook content analyses of these two items [12]. The medical conditions were tabulated and summarised; the number of words in GPs' responses were counted (median and range are reported). Inductive content analysis, in which themes and constructs were derived from the data without imposing a prior framework was conducted [12]. A thematic network was used in analysing GPs' responses by deriving basic themes emerging from the codes given to their words, phrases or sentences [13]. These basic themes were organised into clusters of similar issues, called the organising themes. These organising themes gave rise to an overall global theme, which summarises and makes sense of the clusters of lower-order themes [13].

In reporting our results, GPs' responses are identified by a study identification number after each quotation. An ellipsis (...) is used when words have been deleted. Ethical approval was obtained from the Human Research Ethics Committees at La Trobe University and the University of Melbourne, and the Human Research and Ethics Committees of the RWH. Completion of the anonymous survey was taken as informed consent to participate.

## Results

Three hundred and thirty five GPs responded, giving a response rate of 52% (335/640; 26 potential participants were ineligible). Approximately 76% (253/335) of respondents described the last occasion of decision-making regarding use of medicines in a breastfeeding woman. The median (range) of words used in this item was 19 (2-116). Over one third (37%, 125/335) responded to "any comments about medicine and breastfeeding". To summarise the demographic and personal characteristics of the respondents: 70% of GPs were female; about 37% were in the age bracket of 45 to 54 years; most had obtained their medical degree in Australia (84%); 90% had children; and 49% of GPs or their partners had over 12 months of breastfeeding experience.

Table 1 presents the health issues of breastfeeding women who presented for treatment. The commonest groups of conditions were infections in general (50%, 126/253), of which half were mastitis (24%, 60/253), and depressive disorders (21%, 54/253).

**Table 1:** Health issues in breastfeeding women reported by GPs (n = 253)

Condition	Individual health issue		Categories of health issues	
	**n**	**%**	**n**	**%**

**Infections**			126	49.8
Mastitis	60	23.7		
Other infections	(66)	(26.1)		
Endometritis	11	4.3		
Tonsillitis	9	3.6		
RTI	8	3.2		
UTI	7	2.8		
Nipple thrush	6	2.4		
"Antibiotics"	6	2.4		
Common cold	3	1.2		
Other gynaecological infection*	3	1.2		
Sinusitis	2	0.8		
Giardia	2	0.8		
Miscellaneous infections	9	3.6		
**Depressive disorders**			54	21.3
**Use of analgesics**			13	5.1
**Contraception**			9	3.6
**Low milk supply**			8	3.2
**Atopy**			8	3.2
Hay fever	3	1.2		
Asthma	2	0.8		
Antihistamines	2	0.8		
Urticaria	1	0.4		
Other conditions**			23	9.1
**General comments **(no specific situation)			10	4.0

*v. discharge/vaginitis/pelvic infection

**gastritis/reflux, anaemia, epilepsy, anal fissure, drugs, breast engorgement

Most GPs who cited an infection, especially mastitis, reported the information in a brief, precise manner (median = 12 words, range = 2-46) whereas those who mentioned a depressive disorder tended to write a lengthy explanation (median = 28 words, range = 3-90). Examples of mastitis responses:

• *"Mastitis. Treated with Fluclox [flucloxacillin]. Easy decision on clinical grounds." *(ID 8) (9 words)

In comparison, the description of depressive disorders was wordier and less confident:

• *"Breastfeeding mother with postnatal depression. Discussion took place regarding safety of medication in combination with breastfeeding. Decision was made to try Lexapro [escitalopram] 5 mg daily initially with careful monitoring of the baby and maternal symptoms. No adverse effects were detected. Mother is currently on Lexapro 10 mg, improvement in symptoms with no adverse effects on the baby noted - baby is thriving*." (ID 158) (62 words)

### Emerging themes

Analysis of the 253 responses revealed six organising themes that emerged from the responses: *certainty around decision making; uncertainty around decision-making; need for drug information to be available, consistent and reliable; joint decision-making, the vulnerable "third party"; *and *infant feeding decision*. There was one global theme identified: *complexity in managing risk in prescribing for breastfeeding women *(see Table 2).

**Table 2:** Basic, organising and global themes

Basic themes	Organising themes	Global theme
Comfortable	Certainty around decision-making	
Confident		
Routine		
Happy		
	
Difficult	Uncertainty around decision-making	
Concerned		
Doubt		
	
Accessing available drug information	Need for drug information to be available, consistent and reliable	
Problems with available drug information		
Time-consuming		
Issues with pharmacists		Complexity of managing risk in
		prescribing for breastfeeding women
Involving other health professionals in decision-making process	Joint decision-making	
Involving mother in decision-making process		
	
Risk	The vulnerable "third party"	
Safety		
Exposure to infant		
	
To continue breastfeeding	Infant feeding decision	
To give infant formula		

### Certainty and uncertainty around the decision-making process

The first two organising themes which emerged from the comments GPs made with regard to their decision-making process related to their emotional response. GPs either reported positive feelings which were mainly associated with a certainty about the decision or negative feelings when they were less certain.

#### Positive feelings on decision-making process

The basic themes used to describe positive feelings were *comfortable*, *confident, routine *and *happy*. Of the 60 GPs who reported treating mastitis, 48 (80%) were comfortable with their decision: *"... Mastitis unlikely to resolve without Ab [antibiotic]. Very comfortable." *(ID 300)

GPs were also confident about treating other infections: "*Antibiotic - needed to be suitable for breastfeeding otherwise no concern. Advised re baby effects of no significance*." (ID 131)

#### Negative feelings on decision-making process

GPs reported negative feelings such as *difficult, concern *and *doubt*. These feelings were more evident with prescribing antidepressants than with antibiotics; of the 23 negative feelings, only one related to mastitis, while eight related to depression/anxiety: *"... Concerns about SSRI during breastfeeding by both me and patient. Decision-making process is always fraught and made difficult by conflicting information." *(ID 115) and *"... I think the risks of depression (postpartum) often outweigh the risks of the antidepressants. I felt that there are no right answers to the problem." *(ID 24)

### Need for drug information to be available, consistent and reliable

Before prescribing for a breastfeeding woman, many GPs needed to check sources for information on safety of the medicine; this was not straight forward as sources gave conflicting responses, and sometimes pharmacists' opinion on medicines in breastfeeding was at odds with their own decision.

#### Accessing available drug information

The need to verify the suitability of the drug to be used in breastfeeding women arose in many situations. Sources of information ranged from Therapeutic Guidelines [14], MIMS (a commercial medicines inventory) [15], RWH drug advisory line, pharmacists, specialists (psychiatrists, microbiologist), to Product Information. GPs accessed this information using printed books, online sources, telephone consultations, as well as medical journals and magazines.

*"Depression ... Consulted RWH book, psychiatrist, discussion with pt. [patient] re risk etc." *(ID 199)

*"Considering antibiotics for skin infection. I check product information (PI) on computer to determine safety status of the medication for breastfeeding. I try always to confirm safety on the PI first." *(ID 331)

*"Hay fever. Rhinocort [budesonide]. Avoid antihistamines. Time consuming. Used 2 books + Medical Director [software program] information. Different recommendations re: safety of antihistamines." *(ID 144)

GPs mentioned seeking drug information from pharmacists at several maternity hospitals in Melbourne. These professionals were highly regarded *("The pharmacist at RWH excellent - gives various sources of information and good opinion re: overall management. If not in, she always rings you back - very reliable*." ID 301 Comment).

In the comments section, a number of respondents requested easy access - preferably online - to evidence-based information on the use of medicines for breastfeeding women:

*"I would appreciate ready access to detailed information - books often get misplaced, so internet access would be great." *(ID 60 Comment) and *"... We need a dedicated reliable easy access source*." (ID 164 Comment)

#### Problems with available drug information

Non-availability of easily accessible, evidence based, up to date information on medicines in breastfeeding was mentioned. GPs often mentioned that their sources of information were conflicting and often "over cautious".

*"Depression. Most information is 'personal decision' i.e. no good evidence. Reasons for decision - local psychiatrist opinion, RWH pharmacist's opinion. Difficult finding up to date info." *(ID 152)

*"SSRI in a breastfeeding woman. That it was acceptable. I had to look back at past Australian Doctor [magazine] articles b/c [because] the online sources of MIMS info was too overcautious. OK once I had read the article. I feel able to make an informed decision." *(ID 161)

The process of seeking information was mentioned as time-consuming by three GPs:

*"Depression. Efexor [venlafaxine]. Checked with Box Hill Hospital pharmacist via phone. Got the most reliable and up to date info but the information took hours to obtain. ie. too long." *(ID 321)

Some GPs mentioned wanting more information or that they would have liked guidelines:

*"Antibiotics for mastitis. Decided to use antibiotics + continue breastfeeding/expressing. Mum needed medication. I still lacked clear guidelines as to possible effects on baby + what could have been better option." *(ID 283)

*"A guide similar to preg[nancy] category guide A, B 1,2,3, etc would be useful" *(ID 93 Comment)

Although the questionnaire did not directly address the drug categories for pregnancy, it was evident that there is confusion among some GPs about the appropriate use of these categories. Some GPs incorrectly believed that pregnancy categories could be used to assess safety in breastfeeding:

*"... In general I try to search for a medication that is category A (for pregnancy) & considered safe for breastfeeding"*. (ID 117 Comment)

*"It is easier to get info on pregnancy & medication than it is to get info[rmation] on breastfeeding & medication. If in doubt, I tend to check the pregnancy category. If it is safe in pregnancy, I assume it is safe in breastfeeding...." *(ID 76 Comment)

Several GPs mentioned the need for safety information for complementary medicines: *"We need more access to info[rmation] relating to complementary medicine ..." *(ID 177 Comment)

#### Issues with pharmacists

Several GPs brought up the issue of pharmacists' advice regarding medicines in breastfeeding conflicting with their decision. Two GPs described the pharmacist as *challenging *the GPs' decision:

*"Diflucan [fluconazole] for nipple thrush ... But I have been challenged by pharmacists for this before re. issue of infant exposure, so I don't feel entirely comfortable with it." *(ID 175)

*" ... It was difficult as the pharmacy rang to challenge the Flagyl [metronidazole] use, but I double checked the RWH breastfeeding book & it said it could be used, so we went ahead." *(ID 2)

One GP stated that *"Pharmacist[s] tend to be too conservative and advise against taking anything. Also, they sometimes provide advice against what I say and alarm patients ..." *(ID 246 Comment)

### Joint decision-making

GPs felt that certain situations warranted involvement of several parties in the decision-making process rather than a quick decision on their part. Although this would involve more time and work for GPs, they thought this would help to make a more appropriate and safe decision and increase mothers' compliance with the recommended/prescribed medicine.

#### Involving other health professionals in decision-making process

Seeking advice from specialists was deemed necessary in many instances especially when treating postnatal depression.

*"Had to prescribe an antidepressant. Discussed situation with patient and her psychiatrist ... OK with decision as it involved team care co-ordination ..." *(ID 104)

#### Involving mother in decision-making process

Many GPs discussed the situation and medicines issues at length with the woman herself before arriving at a decision especially with regard to postnatal depression, but in many other instances as well.

*"Postnatal depression. Antidepressant prescribed after long discussion with patient re: prob. areas and current literature/discussion re: safety and proven side effects. I was happy with the decision and I felt the patient was happy." *(ID 22)

*"Acute mastitis. Put on Amoxil [amoxicillin] after D/W [discussing with] Mo. [mother] re relative safety of this antibiotic and need for antibiotic, Mo. initially concerned but happy to take after addressing her issues." *(ID 58)

### The vulnerable "third party"

The fifth organising theme concerned issues of prescribing for one person, the breastfeeding woman, thereby exposing the vulnerable "third party" - the breastfed child - to the effects of the medicine. The basic themes were *risk*, *safety *and *exposure to the infant*.

#### Risk

A general statement such as "benefits outweigh risks" was stated by 15 GPs. In some cases, the GP specifically mentioned considering the risk to the breastfed infant:

*"Recently prescribed Cipramil [citalopram] for postnatal depression to a woman who was breastfeeding. I felt the risk to the baby was low & the drug was important to the woman. I felt reassured that I have been to talks where psychiatrists have said they use this drug in lactating women." *(ID 78)

One respondent added: "*Be careful & know the potential dangers." *(ID 68 Comment)

In some cases, the GP was aware that the breastfeeding woman was more concerned about the possible adverse effect of her medicines on the breastfed infant than the GP was: *"... Patient's reluctance despite reassurance +++. No problem for me, but patient very reluctant to take anything*. "(ID 14) and *"Headache ... Paracetamol 2 tds & r/v [review] ... patient unkeen on medication." *(ID 47)

Some GPs commented that the general public are apprehensive about potential risks with any medicine while breastfeeding ("*Patient concern is very high ..." ID 164 Comment*). One GP suggested that this perception of risk could be negatively affecting breastfeeding rates: *"... public perception is they can't take anything. This may partly be impacting on low uptake of breastfeeding*." (ID 5 Comment)

#### Safety

There were some medicines that GPs regarded as "safe" for breastfeeding women; the examples given were drugs with a longer history of use:

*"Panadol [paracetamol]. To give to her. Safety. Safe and sure*." (ID 287)

*"Gastritis - in patient while breastfeeding? Safety of Nexium [esomeprazole] or Pariet [rabeprazole]. Decided to use Zantac [ranitidine] - older drug more information regarding safety." *(ID 299)

#### Exposure to the infant

Although it could be expected that GPs consider the amount of medicine the infant would receive through the breast milk as an essential part of decision-making, this was rarely alluded to. This is the only quote that refers directly to infant exposure:

*"Sleep difficulties. Advice sleep well hints. Occasional dose of temazepam 10 mg at night and avoidance of overnight feeding to minimize infant exposure. This medicine has relatively short half life. Decision-making process was difficult as lethargy and poor feeding could occur." *(ID 189)

### Deciding how the infant will be fed

In some scenarios it was obvious that the decision whether the mother could or should continue breastfeeding was discussed as a separate issue from the decision about using a medicine.

#### To give infant formula

GPs advised cessation of breastfeeding and the introduction of infant formula in several instances, even in situations that did not warrant such measures:

*"A woman needed Flagyl [metronidazole] for ?anaerobic infection. Information accessed via MIMS Annual (internet). Decided to express + discard for 1 wk + 3 days + formula feed, then resumed thereafter"*. (ID 304)

#### To continue breastfeeding

However, many GPs stressed the importance of continued breastfeeding together with medicines:

*"Pt. on antiepileptic. Continue BF. Checked literature and phoned RWH. Content." *(ID 192)

*"Postnatal depression. Prescribed Zoloft [sertraline] advised to continue breastfeeding. Benefit outweigh risks. I felt Okay with decision." *(ID 138)

### Global theme: Complexity of managing risk in prescribing for breastfeeding women

From GPs' responses about their most recent experience of making a decision concerning a medicine for a breastfeeding woman, it emerged that this was a contentious area, often involving uncertainty and requiring consultation with various colleagues and data sources. GPs were aware that prescribing for a breastfeeding woman leads to the inadvertent exposure of her infant to a potentially harmful medicine, and their role was to manage this risk.

It was also evident that decision-making is a spectrum, from a reflex action - "*there was no decision-making process (ID 76)" *- to a complicated process requiring multiple inputs and consideration. Consensus emerged among respondents that in some conditions, such as infections like mastitis, the decision-making was straight forward and the reports were brief and used positive sentiments, like *comfortable, confident *and *routine*. In these examples, GPs appeared to use their own knowledge and experience and make the decision quickly and on their own. In contrast, GPs found decisions around more complex problems, such maternal depressive disorders, were often time-consuming and difficult. In these examples, a number of processes involving external sources of information may need to be employed: phone calls to specialist doctors or pharmacists, a range of information sources searched and long discussions conducted with the woman and her family.

## Discussion

This study is the first in-depth examination of Australian GPs and their decision-making about use of medicines in breastfeeding women. This paper is based on open-text comments from a survey, which limits our ability to draw conclusions. With only written responses, we were unable to probe the respondents for further explanation for their answers. In some studies, free-text comments may not be representative of all participants: more articulate or less satisfied respondents may bias the response [11,16]. However, three-quarters of our respondents completed the structured question about decision-making, providing us with 253 responses to analyse. Although analysis and reporting of free-text comments are rarely mentioned in textbooks of survey methods or qualitative analysis [16], qualitative analysis can be used with free-text data that are collected in depth [11]. Our findings provide context around how doctors view the issues and have been supported by discussions with GPs - informally and at conferences [17,18].

We found that the overarching theme for these GPs was "*complexity of managing risk in prescribing for breastfeeding women*". Our results confirm the findings of a study conducted among GPs in the north of England, which revealed that the decision-making process of prescribing medicines was regarded as a complex issue due to various reasons such as concerns about drug toxicity and appropriateness of the treatment, and uncertainty about management [19]. The authors found that "prescribing discomfort is a universal, or near universal, experience of prescribers" [19] p. 295. Concern about drug toxicity was the most common reason for GPs' discomfort. So, it appears that prescribing is often an uncomfortable part of any consultation, but this is heightened when prescribing for lactating women. A Danish study found that adopting a conservative attitude and prescribing familiar medicines was one strategy GPs employed to save time and energy as well as reducing the level of uncertainty [20]. A study of GPs' prescribing behaviour in London also found a striking picture of stability, with GPs making very few changes in their prescribing patterns [21].

The concept of risk has become central to our everyday thinking [22], yet a range of cognitive biases can alter our risk perception [23]. Lyerly and colleagues found common patterns in risk perception and reasoning affecting medical decision-making in pregnancy [23]: a tendency to "pursue zero risk to the fetus, independent of the absolute size of the risk, of competing considerations, or of recognition that fetal risk exists in other acceptable contexts" [23] p. 981. Another tendency they identified was that the risks of intervening are given precedence over the risks of *failing *to intervene; for example, maternal medicines for severe asthma may be halted in pregnancy [23]. The same faulty reasoning lies behind the failure to treat lactating women with medicines when appropriate - mother and baby are best served by appropriate medical treatment of the mother and continued breastfeeding for the baby in the majority of prescribing scenarios [8,24].

The public often assumes that all medicines are too risky for breastfeeding women to take. Bellaby explains that responsible parents will avoid an action if they believe there is any risk to their child [25]. The community often believes that a breastfeeding woman must be completely "pure" and her milk absolutely free from contaminants - an impossibility in today's world [26]. In the risk-benefit analysis, the risks of introducing infant formula are rarely considered [27,28]. "It is the physician's obligation not to *eliminate *risk, but to help patients weigh risk, benefit, and potential harm, informed by best scientific evidence and guided by a patient-centred ethic" [23] p. 982.

Risk communication expert, John Paling, states that "... *patients' assessment of risk is primarily determined not by facts but by emotions*" (p. 745) and suggests that doctors remind patients that virtually all treatments are associated with some risk [29]. His advice includes avoidance of descriptive terms "*low risk' *(give numbers, eg. 1 in 10 000) and to offer positive and negative outcomes (eg. how many infants will not have an adverse effect) [29].

It appeared from our study that GPs' perception of risk in prescribing is on a spectrum, from low in certain circumstances to high in others. GPs appeared to make straight forward independent decisions when treating certain conditions such as mastitis and other infections. These decisions seem similar to the "*rules of thumb*" used by Swedish doctors [30]. Our findings confirm those of a study conducted among GPs in Victoria in the mid 1990s, which revealed a similar picture regarding confidence in treating mastitis: approximately three-quarters of GPs reported that they were very confident in treating mastitis in comparison to one quarter for treating postnatal depression [6].

However, this reflex decision-making and lack of reflection, at times led to increased risk - in this case, not for the infant - but risk of poorer outcome for the mother. In some cases, the GP was confident in their management, but prescribed an inappropriate medicine for mastitis (e.g. penicillin [ID 76], amoxycillin [ID 58]). The Australian antibiotic guidelines have recommended a penicillinase-resistant penicillin for at least ten years [31,32]. Although mastitis is a common problem in the postnatal period, it is not always well managed by health professionals [33].

In contrast to the reflex decisions, other decisions required multiple inputs involving much thought and time spent on arriving at the decision. Although this more involved process might help GPs arrive at the most appropriate solutions, in certain instances it did not. GPs reported advising mothers to stop breastfeeding when taking sertraline, metronidazole, and other medicines, although generally these are considered safe for breastfeeding women [34,35].

These poor decisions may indicate a lack of reliable, evidence-based information on the use of medicines for breastfeeding women. Many GPs recognised that product information was overly cautious, that different sources of information gave conflicting recommendations on safety of the same drug in lactating women, and that it took time to gather information on which to base their decisions. Comments on the need for easily accessible evidence-based information supported data from the quantitative part of the survey where 57% of respondents indicated they would prefer a reliable internet database [9].

Although some GPs reported being challenged by community pharmacists, drug information pharmacists were highly regarded. In Toronto, Canada, the Motherisk program provides information about medicines in pregnancy and lactation; 89% of physicians who had called the program commented that the service was very valuable to them [36].

In Australia, as in other countries, drug categories have been created to designate the safety of medicines in pregnancy [37,38]. Similar categories have not yet been created for safety during lactation. The results of this survey, and the interviews conducted with GPs in preparation for the survey, have indicated that many GPs do not differentiate between prescribing during pregnancy and lactation. Concerns about teratogenicity when using medicines in pregnancy are not relevant in the postpartum period, and the amount of medicine transferred to the infant via breast milk is considerably less than that transferred through the placenta to the fetus [8]. GPs in this study did not mention the factors considered important when pharmacologists consider risk-benefit analysis: drug transfer into milk, dose regimen and infant age [8], suggesting that education in this area could be beneficial.

Recently, the Food and Drug Administration in the US has suggested major revisions to the physician labelling for prescription drugs to provide better information about the effects of medicines used during pregnancy and breastfeeding [39].

There are several limitations to this study. It is likely that GPs more interested in the topic have responded to the survey, but we had a sample of 253 GPs, with over a 50% response rate to the survey and over 75% completing these open comment items. If other GPs were less interested in this issue and less confident in their management of postnatal issues requiring medicines, then our results may lead to an underestimation of the actual problem. Our respondents used a range of sources of information, which reflected their familiarity with prescribing for breastfeeding women. GPs not associated with the RWH would be likely to be less familiar with the RWH *Drugs and Breastfeeding *book [35] or hospital pharmacy telephone advice service. Although other sources of evidence-based information are available [40], it is likely that GPs who do not provide shared maternity care would be less familiar with them.

Although we are not able to accurately determine the prevalence of these postpartum conditions, the frequencies reported indicate which conditions were commonly encountered for treatment among the respondents, and were similar to those found previously in Australia [5].

Analysis of written text does not allow an in-depth iterative approach to deeper discovery of meanings. Some GPs' responses were not clear cut and clarification was not possible in this study. The structured nature of the open-text comments - and the large number - enabled an analysis that we feel is robust, but which needs to be further explored in qualitative research in the future.

## Conclusions

The decision to prescribe medicine for a breastfeeding woman is not always easy or simple. Doctors need to manage the risks by balancing the need to treat the mother for a medical condition and concurrently support breastfeeding of the infant. The public has great concerns about taking medicines while breastfeeding. Guidance for making the appropriate decision in the form of evidence based clear guidelines and online databases are not always available or readily accessible. Current available information may actually be contradictory, thus contributing to the complexity of decision-making for GPs. Therefore in many instances GPs are faced with great difficulties, even with commonly prescribed medicines like metronidazole. Hence, at present GPs believe that many decisions are "personal decisions" rather than evidence-based and they feel a need for easily accessible evidence based clear guidelines available in print as well as electronically on prescribing medicines for breastfeeding women.

## Competing interests

The authors declare that they have no competing interests.

## Authors' contributions

HSJ conducted the content analysis with input from LHA and MVP. LHA and MVP designed the project and obtained funding. All authors contributed to writing the paper.
